# Quality of services in health education nurse-led clinics: an Iranian service providers and service recipients experience

**DOI:** 10.1186/s12913-024-11057-1

**Published:** 2024-05-03

**Authors:** Zohre Pouresmail, Fatemeh Heshmati Nabavi, Maryam Rassouli

**Affiliations:** 1 Nursing and Midwifery Care Research Center, Mashhad University of Medical Sciences, Mashhad, Iran; 2 Department of Community Health and Psychiatric Nursing, School of Nursing and Midwifery, Mashhad University of Medical Sciences, Mashhad, Iran; 3 Department of Medical-Surgical Nursing, School of Nursing and Midwifery, Mashhad University of Medical Sciences, Mashhad, Iran; 4 School of Nursing & Midwifery, Shahid Beheshti University of Medical Sciences, Tehran, Iran

**Keywords:** Practice patterns, Nurses, Qualitative research, Health Education

## Abstract

**Background:**

Patient education is a vital role of nurses in nurse-led clinics(NLCs). Since 2011, independent NLCs entitled health education Nurse-led clinics(HENLCs) have been established in Iran. In order for this newly developed service to be able to perform perfectly in implementation and evaluation, it should be explained based on one of the quality evaluation models. The objective of the study was to determine the dimension of service quality in HENLCs based on service providers’ and service recipients’ experience.

**Methods:**

This research is a qualitative study of directed content analysis type conducted between May and November 2020. Twenty-nine participants who had rich experiences in the patient education in HENLCs were interviewed in this study. Asarroodi et al.’s (2018) qualitative content analysis method was used for data analysis, and MaxQDA software was used for data management. We used credibility, dependability, and Confirmability to confirm the trustworthiness of the study’s findings.

**Results:**

In this study service providers including managers, policymakers, decision-makers, nurses, physicians, and service recipients including patients and families participated. Seven generic categories, including (1) a competent and self-motivated nurse educator, (2) an easily accessible and comfortable environment, (3) informational-educational materials and health education equipment, (4) motivational facilities, (5) access to the health education support team, (6) organizational communication supporting the education process, and (7) receiving the patient education fee, constituted the main category of structure. Five generic categories, including (1) assessment and determination of the educational needs of the target group, (2) description of the nurse’s duties, (3) teaching-learning methods, (4) patient referral, and (5) the process of preparing and publishing educational content, constituted the main category of process. One generic category called evaluation constituted the main category of outcome.

**Conclusion:**

Based on the results of this study, it is suggested to managers to pay attention to the dimensions of the quality model of Donabedian (SPO) in setting up and developing the performance of HENLCs, it is recommended that future quantitative studies based on the categories formed in this study evaluate the observance of the dimensions of structure, process and outcome.

## Background

Nowadays, trends, such as increased health literacy of clients, shortened hospitalization, and increased prevalence of chronic diseases related to lifestyle and aging, have culminated in making the role of patient education vital for patient care and developing care from inpatient to outpatient wards [1–4]. One of the outpatient centers in which nurses provide services is a nurse-led clinics (NLCs). NLCs are centers that increase nurses’ autonomy and professional level by providing independent nursing services since the 1980s [5]. These centers have been launched in Sweden, England, America, New Zealand, Canada, and Australia [6]. Many nurses in these clinics provide health and specialized care services independently in the community or hospital outpatient wards [5, 7].

The necessity of attention to the learning requirements of patients and the role of education in improving patient outcomes, particularly for chronic and outpatient patients, culminated in the launching of independent NLCs in 2010 in Iran to provide education and counseling services to patients in hospital outpatient wards of big cities of Iran. The provision of services in these centers has been voluntary and creative. Given that the educational role of nurses is expanding, starting and continuing the activities of these clinics has always faced barriers. Some of these barriers are regarding not defining the position of these centers in the hospital organizational structure(8), lack of independence in providing services, and difficulty providing human resources [9].

Following the international trends occurring at the international level and the development of patient education from hospitals to outpatient centers and home and community care, in 2019, the development and promotion of patient and family education programs became the research priorities of the Nursing Deputy of Ministry of Health, and in June 2022, the “executive instruction of nurse-led clinics for patient education and follow-up” was officially announced to the whole country by the Nursing Deputy of Ministry of Health, Treatment and Medical Education [10].

In order for this newly developed service to be able to perform perfectly in implementation and evaluation, it should be explained based on one of the quality evaluation models. Donabedian’s (1966) Structure-Process-Outcomes (SPO) conceptual framework was used to examine health services and evaluate the quality of care [11].

establishing NLCs for patient education in hospital outpatient settings entitled Health Education Nurse-led Clinics (HENLCs) in some centers has been successful. However, it is required to further investigate the requirements and necessities for setting up and continuing the activities of these clinics, which should be paid attention to by policymakers in the rules, in the areas of structure, process, and outcome of providing this service. This qualitative study is part of a larger mixed study, in which 46 standards (13 structure, 28 process and 5 outcome) were developed for setting up HENLCs [12] and the results of which were used in designing the “executive instruction of nurse-led clinics for patient education and follow-up.”

At the international level, many nurse-led clinics have been set up [13–15] in which the experiences of providing and receiving services in them need to be investigated.

In Iran, various studies have also investigated the effectiveness of patient education in hospital ward settings [16–18]. However, few studies are focusing on patient education in outpatient centers. For example, in one study, researchers examined the characteristics of patient education in home care centers [19] and Another study has focused on the topic of patient education rooms in hospitals [20]. Recently, HENLCs have been established in Iran. As qualitative approaches provide a rich and deep description of the participants’ experiences and their context, the qualitative content analysis method was used to explore the experience of providing and receiving self-care education and counseling services in HENLCs.

The objective of the present qualitative study was to determine the dimension of services quality in HENLCs based on service providers (nurses, nursing managers, and center managers) and service recipients(patients and their families) experience in Iran.

## Method

This is a qualitative study of directed content analysis conducted in Mashhad hospitals between May and November of 2020. Based on Asarroodi et al.’s (2018) inductive content analysis method, the preparation phase was performed by going through seven stages including acquiring the necessary general skills, selecting the appropriate sampling strategy, deciding on the analysis of manifest and/or latent content, developing an interview guide, conducting interviews and transcribing interviews, specifying the units of analysis, and being immersed in the data [21].

In order to conduct this study, service providers including managers, policymakers, decision-makers, nurses, physicians, and recipients of patient education (patients, families), were selected through purposive sampling and were selectively entered the study. To do this, 29 participants who had rich experiences regarding providing or receiving patient education in HENLCs were selected and interviewed. The researcher selected people who had rich experiences in providing and receiving patient education in HENLCs. Considering that second author was the secretary of the patient education committee of Mashhad University of Medical Sciences and the Ministry of Health, the identification of people with rich experiences was facilitated. The first author was involved in setting up the first HENLCs. The third author was one of the policymakers of patient education in the Ministry of Health, Treatment and Medical Education of Iran. The interviews were conducted in a quiet room in the hospital that was agreed upon by the interviewer and the interviewee. In the providing service group, four educational supervisors, five health education supervisors, two nursing managers, and two hospital managers four nurses, three physicians, one patient education expert in the deputy of treatment, and two nursing faculty members were interviewed. Finally, in the receiving patient education group, two patient families and four patients referring to the HENLCs were interviewed (Table 1).

**Table 1:** The participants’ characteristics in the qualitative part of the study

Participant Code	Age (Year)	Gender	Work Experience in Health Education Clinic (Year)	Education Degree	Position	Field of Study
1	39	Female	6 years	Master	Educational supervisor	Nursing
2	40	Female	5 years	Master	Educational supervisor	Nursing
3	45	Female	6 years	Master	Health education supervisor	Nursing
4	55	Female	7 years	Bachelor	Nurse	Nursing
5	40	Female	6 years	Master	Educational supervisor	Nursing
6	46	Female	5 years	Master	Educational supervisor	Nursing
7	34	Female	6 months	Bachelor	Nurse	Nursing
8	56	Female	6 years	Master	Nursing manager	Nursing
9	51	Female	3 years	Bachelor	Nurse	Nursing
10	50	Female	7 years	Doctorate	physician	Medicine
11	48	Female	4 years	Master	Health education supervisor	Nursing
12	45	Female	5 years	Master	Nurse	Nursing
13	35	Female	-	-	Patient referring to the clinic	-
14	46	Female	7 years	Doctorate	physician	Medicine
15	48	Male	3 years	Master	Nursing manager	Nursing
16	49	Female	-	Master	Health education supervisor	Nursing
17	48	Female	2 years	Doctorate	physician	Medicine
18	37	Female	7 years	Master	Health education supervisor	Nursing
19	31	Female	1 year	Bachelor	Nurse	Nursing
20	54	Male	4 years	Doctorate	physician	Medicine
21	39	Female	7 years	Master	Expert in the deputy of the department	Nursing
22	38	Female	-	Diploma	Patient	-
23	44	Female	-	Elementary School	Patient companion	-
24	55	Female	-	Elementary School	Patient companion	-
25	45	Female	-	Elementary School	Patient	-
26	31	Female	-	Diploma	Patient	-
27	55	Male	2 years	Master	Faculty member	Nursing
28	40	Female	2 years	Doctorate	Faculty member	Nursing
29	46	Male	4 years	Doctorate	Hospital manager	Medicine

The inclusion criteria for the group of service providers included having a history of policymaking or participating in launching HENLCs, a clinic being active in their hospital, and managers participating in patient education programs, participants in providing patient education to the patients in HENLCs and participants in preparing and reviewing patient education contents and media.

The inclusion criteria for the group of recipients of patient education included patients or families who had received patient education once at the HENLCs and returned to this center again and were over 18 years old. The exclusion criteria were unwillingness to record audio and unwillingness to participate in the study.

An in-depth, individual face-to-face interview and semi-structured interview was conducted using the interview guide by the first author of the study in the HENLCs in hospitals of the city of Mashhad, and the process continued until information redundancy and data saturation, i.e., until no new information was obtained through the following interviews and the collected data were the repetition of the previous data, no new codes were obtained, and the categories reached conceptual saturation [22].

The main axis of the dialogue in these interviews was the participants’ experiences of providing or receiving patient education in HENLCs. The interview guide was written based on Kallio et al.’s. (2016) study [23] and consisted of open-ended questions according to the study purpose and targeted questions regarding the main categories in the Donabedian model (Table 2). The interviews were recorded by two recorders, immediately transcribed as texts, and then analyzed using the inductive qualitative content analysis method based on the Donabedian model.

**Table 2:** Open-ended questions according to the study purpose and targeted questions regarding the main categories

Participants’ Group	Type of Questions and Categories		Question
Provider of patient education (managers, nurses and doctors)	Open-ended questions according to the study purpose		• Explain your experience regarding policymaking and patient education in the self-care clinic?
	Targeted questions regarding the main categories	Structure	• Was the space devoted to the education appropriate? What characteristics should an appropriate space have? • How was patients’ accessibility to the clinic? • How do the nurses you chose as managers and executors for self-care clinics and patient education activities gain your trust (what characteristics do the nurses have that make you trust them? ) • How did you devote facilities and equipment to launch a health education nurse-led clinic?
		Process	• How is the patient referral to the clinic? • How has the doctor-nurse relationship been established? • By what process do you educate your patients? • What has made patients trust you? • How does the physical environment of the self-care clinic affect the patient outcomes? • How do you supply the costs of launching and operation of self-care clinics? • As a manager, how do you attract the cooperation of physicians and other members of the treatment team to participate and refer patients to the self-care clinic?
		Outcomes	• What makes patients come back to you again? • In your patients’ opinion, meeting what needs by your education is good? • How did you notice that your education was effective? • How do you evaluate the effectiveness of the education or the performance of the health education nurse-led clinic? • What are the effective indices of performance in nurse-led clinics that you consider in your work?
Recipients of patient education (patients, families)	Open-ended questions according to the study purpose		• Explain your experience of receiving education from nurses in the self-care clinic?
	Targeted questions regarding the main categories	Structure	• What were the characteristics of the environment in which you received education? • What characteristics did your nurse educator have that made you trust her words? • How did you access the health education clinic?
		Process	• Did you use the education that the nurses provided to you? • What were the characteristics of the education you applied? • During the education you received, how were the recommendations that you took seriously and implemented provided? • How were the doctor’s explanations that could guide you? • How was the doctor engaged in your education? • How did the family participate with you in receiving education?
		Outcome	• Which of the recommendations and education of the nurses resulted in your health improvement?

Asarroodi et al.’s (2018) qualitative content analysis method was used for data analysis, and MaxQDA software was used for data management [21]. The analysis process is performed in inductive and deductive content analysis in three main phases, including preparation, organizing, and reporting [24]. At this stage, after transcribing each interview and considering its transcribed text as the unit of analysis, each text was read several times until the data immersion occurred. During this stage, the answer to these questions was always taken into consideration by the researcher: What event is happening? Who is speaking? Where is it happening? When did it happen? What is happening and why?

Based on Asarroodi et al.’s (2018) content analysis method, the organizing phase consisted of developing a formative categorization matrix, the theoretical definition of the main category and subcategories, determining coding rules for the main category, pre-testing the categorization matrix, choosing and specifying the anchor samples for each main category, performing the main data analysis, the inductive abstraction of the main categories from preliminary codes, and establishing links between the generic categories and main categories [21]. The researchers, in the organizing phase, created a constrained matrix for analysis. In this matrix, the creation of new main categories is not allowed. The data were reviewed several times to find content that matched predefined categories or could be a sample for them, and preliminary codes were assigned to them. Afterward, the stages of grouping, categorization, and abstraction were performed so that the generic categories were created, and the possibility of placing these generic categories in the main categories in the matrix was then examined conceptually and logically [25].

To ensure the credibility of the findings, a prolonged eight-month engagement in the field, member check, peer check, and full recording and transcribing of the interviews were carried out immediately after each interview. Data dependability was provided by presenting preliminary codes obtained from the analysis of the participants’ experiences and the abstraction stages to the experts and conducting an audit by them. Confirmability was taken into account in such a way that the texts of several interviews and generic codes and categories were submitted to the second and third authors and to two experts who were acquainted with the way of analyzing qualitative research data but were not the research team members, and the data analysis process was therefore assessed and confirmed. Finally, the researcher observed authenticity by describing the participants’ experiences of providing or receiving patient education in HENLCs and by showing different realities.

In order to conduct the research, permission was obtained from the Regional Ethics Committee in Medical Sciences Research (IR.MUMS.NURSE.REC.1398.057), and written consent was obtained from all participants.

## Results

Results were obtained from 29 interviews. Table 1 shows the demographic characteristics of the interviewees. All generic codes and categories constituted the main categories in the matrix, including structure, process, and outcome. Data analysis led to the extraction of 3 main categories and 13 subcategories (Table 3).

**Table 3:** The main categories, generic categories, and subcategories created in the qualitative stage

Subcategories	Generic Categories	Main Category
competencies	A Competent and Self-Motivated Nurse Educator	Structure
Meta- competencies		
Characteristics of the health education clinic room	An easily accessible and comfortable environment	
Availability for doctors and nurses		
Availability for patients		
Clinic introducing equipment	Informational-educational materials and health education equipment	
Non-educational and non-consumable equipment		
Equipment related to providing care services to patients		
Educational media		
Motivational facilities	Motivational facilities	
Resident doctor	Access to the health education support team	
Clinical psychologist		
Proficient staff to prepare teaching aid materials and educational media		
Participation of managers in launching and operation of outpatient nurse-led clinics	Organizational communications supporting the education process	
Receiving education fee	Patient education fee	
Free education and not receiving fee		
Target group	Assessment and determination of the educational needs of the target group	Process
Educational need		
Nurse’s duties regarding patient education	Description of the nurse’s duties	
Nurse’s duties regarding following up the educated patients		
Nurse’s duties regarding designing educational content		
In-person and remote education	Teaching-learning methods	
Individual and group education		
Referral by the doctor for outpatients	Patient referral	
Referral by the nurse for patients being discharged		
Participants in preparing educational content and materials	The process of preparing and publishing educational content.	
Characteristics of educational materials		
Evaluation of patient’s outcomes	Evaluation	Outcome
Evaluation of nurse’s performance		
Evaluation of outcomes related to clinic performance at the organizational level		

### The main category of structure (general mobilization of facilities and human resources in the organizational structure)

Structure describes features and attributes of the centers in which care takes place [26]. According to Donabedian, the structure of each care provider system is adjusted based on the service provision type, and the structure type can affect the service providers’ behavior and, consequently, the quality of care provided [26]. In this study, in the main category of structure, the generic categories created from qualitative data include (1) a competent and self-motivated nurse educator, (2) an easily accessible and comfortable environment, (3) informational-educational materials and health education equipment, (4) motivational facilities, (5) access to the health education support team, (6) organizational communication supporting the education process, and (7) receiving the patient education fee (a motivating factor for the patient or a barrier to receiving education).

#### A competent and self-motivated nurse educator

The participants believed that a nurse could play the role of an effective educator in HENLCs with competencies such as having adequate knowledge and experience, being a role model, effective professional relationships, effective communication skills, skills in providing patient education, and teamwork skills and meta-competencies such as commitment to performing duties, moral personality, mastery of the English language, creativity, critical thinking, interest, motivation, and computer skills:

*“We must have interested and scientific colleagues, who can proceed with the work…, have an educational view, be experienced, have good communication skills…”* (p20).

*“Ms. … was really perfect; she spoke very sympathetically as if one of her own family members was affected by the disease… Ms. … gained my trust from the very first moment I saw her…”* (p23).

#### An easily accessible and comfortable environment

Based on the participants’ experiences, the characteristics of the environment allocated to the HENLC play a significant role in the referral of patients and their families and repeated referrals. The easy accessibility of HENLC for physicians and nurses meant being close to the specialized ward and physicians’ access to the clinic. The easy accessibility of the HENLC for patients meant its placement in the main hall of the clinic for outpatients’ easy access to it. The participants’ experiences denoted that the presence of a separate room specific for patient education, a waiting room, and an appropriate place for group education, and also features, such as proper lighting, silence, simplicity, and proper ventilation, can extend the patient length of stay in the HENLC to receive education:

*“I remember feeling suffocated in that room; perhaps, my status was not good, but I exactly remember that the room was dark, the ventilation did not work well, it was hot and humid, and there were no windows, and it made me feel suffocated; so, I liked to go out quickly…”* (P22).

#### Informational-educational materials and health education equipment

Considering the participants’ experiences, since the HENLCs had been launched recently, the presence of a clinic introducing equipment, such as banners and stands, was necessary to attract the clients’ attention. Moreover, the presence of non-educational and consumable equipment, such as office facilities and supplies, heating and cooling facilities, communication devices, and equipment related to providing care services to patients, such as glucometers and special chairs for breastfeeding, was necessary. Furthermore, given that the main activity of these clinics was providing patient education, the presence of educational media such as educational posters, educational booklets, simple educational videos, pamphlets, and translated educational contents for international patients in HENLCs was necessary.

*“Yes, I remember; the room had a big banner; it was a small room, but it had a big banner. I found the room through the banner.”* (P26).

*“We were supposed to prepare some equipment: A glucometer kit, a glucometer, a sphygmomanometer… the table on which our colleague was going to work, an examination bed, a divider… books, pamphlets, and educational videos… all were necessary for patient education.”* (P18).

#### Motivational facilities

The participants’ experiences showed that given the newly emergent nature of patient education in HENLCs, supplying incentives whose provision was important for patients and families, such as helping make an appointment to see the physician, the possibility of doing a test without long waiting time, and receiving educational gifts such as glucometers and books can raise the motivation to refer for receiving patient education.

*“In my opinion, it would be really helpful to give the patient and his/her companion an advantage in the educational clinics; for example, a simple blood glucose test with these blood glucose samples they take; for example, a blood pressure control or BMI calculation, …”* (P20).

#### Access to the health education support team

Based on the participants’ experiences, in addition to a nurse educator, HENLCs need proficient and experienced human resources to support the health education process. These members consisted of resident physicians and available physicians, clinical psychologists, and proficient staff to prepare teaching aid materials and educational media.

*“It is good for us to ask a psychologist for help who will be present in the hospital for at least two or three days. … The patient who is going through the last stages of the illness and is in the hospital along with his/her companion for about twenty days or one month receives supportive treatment, needs more psychological support…”* (P12).

#### Organizational communication supporting the education process

According to the participants’ experiences, organizational communication from the senior management of the hospital to the lowest levels of the organization must support the patient education process. In other words, starting and continuing the effective activities of health education nurse-led clinics requires supplying resources, equipment, and human resources. Supplying these resources in the hospital’s competitive and demanding environment requires the constant support of the organization’s senior managers.

*“…Look, the hospital manager’s support is very important, i.e., the person who is at the top should have an educational view; this is essential for establishing the nurse-led clinic”* (P8).

*“At first, we started almost from scratch; we even did not have an ordinary room, but it was an advantage that the nurse manager was very persistent and supportive, and she was able to allocate a room for this work with her persistence…”* (P5).

#### Receiving the patient education fee (a motivating factor for the patient or a barrier to receiving education)

There was a disagreement between the participants regarding receiving the education fee from the patient. Some underlined the positive facets of receiving a minimal fee from the patient for receiving educational services from the NLC, and some disagreed with it. Based on the experiences of some of the participants, receiving the patient education fee culminates in patients’ more attention to education and perceiving the value of patient education, and provides the costs of services, such as preparing educational videos and pamphlets, whereas based on the experiences of some other participants, receiving the patient education fee causes patients to get away from education. In this regard, the participants’ experiences were as follows:

*“We must be careful not to impose costs on the patient regarding patient education at all; even if we have a shortage, we should not decide to educate the patient and take money from him/her. This is not true at all, i.e., in my opinion, it should be stressed that this duty should not impose a financial burden to the patient.”* (P29).

*“In my opinion, just as a fee is defined for everything…, a specific fee should also be defined for patient education so that the patient pays it; now, we can say that if the patient cannot afford the fee, he/she should go and ask for help from social workers but the patient being able to afford should pay it so that he/she knows that the education is being provided is important. I am not an idle person to come and sit here; if the fee is defined, individuals will feel its importance.”* (P28).

### The main category of process (identifying and meeting the needs accurately)

Process refers to the activities that take place between providing and receiving care(41). In other words, process means the activities consisting of health care -including diagnosis, treatment, rehabilitation, prevention, and patient education- which are typically carried out by professional personnel (41). In this study, in the main category of process, generic categories of qualitative data include (1) assessment and determination of the educational needs of the target group, (2) description of the nurse’s duties, (3) teaching-learning methods, (4) patient referral, and (5) the process of preparing and publishing educational content (Table 3).

#### Assessment and determination of the educational needs of the target group

The participants’ experiences indicated that chronic patients with heart diseases, diabetes, cancer, and hypertension, newly diagnosed patients, and patients who need special care after discharge need to receive education regarding nutrition, taking injectable and non-injectable medicines and their complications, self-care education at home, connection care, education regarding mental health, and activity education based on the educational needs recognized by the nurse.

*“The first thing we discussed, I remember, they explained very well that the patient might experience hair loss, become like this, like that, which finally they said the patient should wear a hat…; about what to eat, … they said you should prepare cool and soft foods because the patient’s mouth might get sore…”* (P23).

*“I think that patients receive education in many fields; for example,…, nutritional care in patients with, nutritional care and physical activities for patients with heart attacks and heart failure. Many of these are also very, very, very important and are the bases of treatment…”* (P20).

#### Description of the nurse’s duties

Based on the participants’ experiences, the nurses working in the HENLC do three main tasks, which involve the nurse’s duties regarding patient education, including obtaining demographic information and patient history, patient education, getting education feedback from patients, registering education in a specific registration form and evaluating them, and following up the educated patients, and the nurse’s duties regarding designing educational content such as preparing educational content, engaging in content localization, and making the educational content applied.

*“Sure… the education is repeated, in which the person comes and asks; we really do a test exactly at that time; for example, we say at the end that ‘What kind of food should he/she eat?’ or ‘When should he/she take the medicine we told him/her?’ We get feedback from the patient right there.”* (P27).

*“We needed to localize many of the hematology pamphlets. Ms. …, who was also a nurse educator in the clinic, commented on them and said that, for example, it would be better to write this sentence here instead of that sentence. She had worked with various doctors and knew how the pamphlets would be more practical.”* (P1).

#### Teaching-learning methods

The participants’ experiences indicated that nurse educators in the HENLC chose their teaching method based on the conditions, available facilities, and patients’ number and type from among in-person, remote, and group and individual methods.

*“It was very good given that she educated one by one and sent each person inside and taught individually.”* (P24).

*“Group education is excellent… For example, concerning the ostomy, an individual who had received an ostomy one year ago, someone who had received an ostomy recently, and a person who had received an ostomy two weeks ago all of us were put together in the same group. They helped each other”* (P27).

#### Patient referral

Based on the participants’ experiences, a critical part of the care provision process of HENLCs is how to refer patients to these clinics. Since playing the nurse’s educational role independently and in the location of HENLCs without playing the caring role for patients is a newly emerging and developing phenomenon in hospitals, patients need to be informed and approved by the trusted members of the care team in facing this new service in a new location. Referral by the physician or the caring nurse in the ward creates this trust and confidence in the patient for the first referral. Since HENLCs provided their services to outpatients and chronic inpatients at the time of discharge, from the participants’ perspective, referral by a physician for outpatients and referral by a caring nurse for patients being discharged were two successful referral methods. Introducing the new education and counseling service in NLCs was carried out through methods, such as informing in inpatient wards, patient follow-up SMSs, occasional programs, and via other clients and patients.

*“We had patients who went to the physician to be visited, and then the physician sent them to our room so that we educate them. These patients came more seriously; sometimes, they even waited for a long time in the hall for the room to get empty.”* (P12).

*“Then, we prepared a form in which the* physician *wrote the patient’s name, surname, and specifications, and then wrote the education needed by the patients in the referral sheet…”* (P6).

#### The process of preparing and publishing educational content and materials

The participants’ experiences indicated that planning should be done to prepare and publish educational content and materials. Educational contents and materials will be practical and usable if they are prepared as a team with the cooperation of physicians, nurses, and faculty members and also using valid and up-to-date references. Besides preparing educational materials, planning should also be done concerning the way of publishing and making the materials available to patients. The participants’ experiences indicated that when the educational contents and materials were prepared by performing a needs assessment and determining the target group, adding valid references, identification number, support organization, and the clinic’s phone number and work hours, they would be approved by all team members and would be practical. Furthermore, adding contact information could motivate patients for future referrals.

*“For our pamphlets and media, we always tried to use our specialist physicians in this field; it means, for example, if our colleagues prepared a pamphlet, we would ask a female specialist physician to have a look at the pamphlet, correct any point more or less, if any, and sign and confirm it…”* (P29).

### The main category of outcome (program and learner evaluation)

Outcome refers to pleasant or unpleasant changes attributed to the care in individuals and populations, indicating the effectiveness and quality of the care provided [26]. In this research, the participants believed that it is necessary to evaluate the performance of NLCs in three areas, including evaluation of patient outcomes, evaluation of nurse performance, and evaluation of outcomes related to clinic performance at the organizational level. Based on the participants’ experiences, evaluation is one of the necessary components for improving and promoting patient education programs in HENLCs. The evaluation of outcomes related to clinic performance at the organizational level included the number of clinics referring to the clinic, patient satisfaction, referrals to the emergency ward because of not receiving appropriate education, and the quality of patient education, the evaluation of nurse performance included assessing recorded reports and in-person visits, and the evaluation of patient outcomes included getting the education feedback from patients and patient follow-up a few days after referral.

*“… Well, first of all, when something starts, you do not expect to gain the results very soon; a period must pass, but we evaluated the performance of the clinic based on the recorded performance, the patients referring, the way of education, and the things that were recorded.”* (P29).

## Discussion

The objective of the study was to determine the dimension of service quality in HENLCs based on service providers’ and service recipients’ experience. The results indicated that in structure domain, a competent and self-motivated nurse educator, an easily accessible and comfortable environment, informational-educational materials and health education equipment, motivational facilities, access to the health education support team, organizational communications supporting the education process, and patient education fee determination were needed to stablish a HENLCs.

Nurses need competencies and meta-competencies to work in NLCs. In this regard, a review study suggested common themes in the standards of nurses’ competencies in primary healthcare centers as clinical performance, communication, professionalism, health promotion, teamwork, education, research, evaluation, and information technology [27]. meta-competencies are high-level skills and capabilities that are shaped based on competencies and culminate in learning, adaptation, prediction, and creativity [28]. Another finding of the study was specifying an easily accessible and comfortable environment, informational-educational materials and health education equipment, and motivational facilities. Specifying these cases depends to some extent on the organizational culture in Iran. The organization’s success in achieving its goals depends on the organization’s structure and culture and its management strategies [29]. Organizational culture contains dimensions such as attention to detail, power distance, ambiguity aversion, risk-taking, adaptability, innovation, individualism, being team-oriented, being outcome-oriented, cohesion, and short-term versus long-term attitude [29].

Based on studies, organizational structure and culture can affect the moral performance of nurses in Iran [30, 31]. Therefore, specifying details in the organizational culture can contribute to the success of HENLCs.

According to the results of the present study, attention to details, such as determining the features and dimensions of the physical space, equipment, and facilities in Iran, is important. In similar studies, it has been shown that the lack of attention to details, such as the location of the clinic, can lead to failure in achieving the goals [32]. Another finding of the present study concerning the structure of NLCs was the determination of access to the health education support team and organizational communication supporting the education process. In this regard, it was shown in the study in Australia that a successful NLC was formed by a support team (medical and nursing supervisors and healthcare workers), organizational managers, and the hospital manager and executive manager and treatment center [32]. One of the challenging issues discussed in this study was receiving the patient education fee, with which some agreed and some disagreed. Proponents of receiving the patient education fee believed that paying the minimum fee for patient education was a reason for making patients and families appreciate receiving educational services and further adhering to education. On the other hand, the opponents acknowledged that receiving the patient education fee could cause patients to further move away from receiving educational services. The research results show that patients’ poor treatment adherence culminates in not improving their outcomes, and subsequently, increased use of medical services and increased care costs, and imposing more costs on the patient [33]. Therefore, it is logical to receive the patient education fee to save time and treatment costs.

Regarding the patient education process in HENLCs, based on the participants’ experiences, it was found that the process of assessing and meeting the needs accurately must be specified, so that patient education services in HENLCs are provided successfully. These cases, similar to the themes identified in the area of structure, refer to determining the service details in the organizational culture. Some studies suggest that assessing the patients’ learning needs, personalizing the patient education content, making education patient-oriented instead of disease-oriented, and evaluating the patient’s perception of education are some areas of the education process that must be taught to nurses [24, 34, 35]. Successful implementation of patient education relies on effective planning [36]. One of the themes specified in this study was patients’ trust in nurses’ education through referrals from trusted members of the treatment team (physicians and nurses of inpatient wards). In the Iranian community, physicians have a special scientific and professional position, and the recommendations provided by them are of importance to patients and families. This issue has been proposed in one study entitled the physician’s authority in education. Accordingly, it has been stated that physicians can have a significant contribution to creating a sense of trust among patients and families in the education provided by the nurse and making the nurse’s educational role valuable [37]. Therefore, it can be said that although the professional employees of the NLC are nurses, the cooperation of physicians in patient referral can greatly help develop the services of NLCs.

Another issue mentioned by the participants in the present study was “the process of preparing and publishing educational content and materials.” In a study, the stage of preparing educational materials has been mentioned to include how to determine the title, individuals participating in the process, the competence of participants in the process of preparing educational materials, related standards, patient feedback (if any), and outcomes, printing facilities, the process of revising or updating, and reviewing educational materials. In this method, educational brochures are prepared for educating patients and families supported by the senior physician or his/her assistant, the nursing manager, the quality improvement manager, two members of the quality improvement team, the physicians managing the internal and surgical wards, and the managers’ administrative assistants. Then, the educational materials are evaluated based on the criteria for preparing the health quality standard brochure [38, 39]. Going through a specific process for preparing educational materials causes trust in the preparation of standard and practical educational content.

In addition to the above findings, the results indicated the importance of paying attention to evaluation in the area of outcome. Evaluating the performance of NLCs should be performed in three areas, including evaluation of patient outcomes, evaluation of nurse performance, and evaluation of outcomes related to clinic performance at the organizational level. In this regard, one study has suggested evaluating nurse-led clinics in three parts: The effect on the patient, the effect on the service or the organization providing the service, and the effect on nursing. Regarding the effect on the patient, continuity of care, promotion of health outcomes, satisfaction, and quality of life; regarding the effect on service provision/ the organization providing service, the difference between the professional groups providing the service, the influence on resources, safety and acceptability, resources of referral to NLCs, the number of patients visited, the influence on waiting time, duration of consultation, outcomes compared to performance guidelines or available national indicators such as referrals to other organizations and discharge; and regarding the effect on nursing, the educational needs of the position holder, registered activities such as the number of prescriptions, the number of referrals to other medical professions, the influence on other role functions, and responses from physicians have been mentioned [5].

### Limitations

Our study had several limitations. First, most of our study participants were female because most nurses are female in our healthcare system. Second, at the beginning of the COVID-19 pandemic, the activities of HENLCs were limited, and access to patients for interviews was difficult. However, all patients and caregivers participating in the qualitative phase were female; data saturation was the criterion for the end of sampling.

### Practice implications

Identifying the experiences of providers and receivers of patient education in HENLCs can help nursing managers in improving the quality of services.

## Conclusion

Setting up and developing HENLCs based on the experiences of recipients and providers of patient education and based on the Donabedian quality model (SPO) can facilitate success in performance and achieving the goals of these clinics. Patients’ access to self-care, prevention of complications of chronic diseases, prevention and screening of chronic diseases, reducing the length of hospital stay and reducing readmissions are among the goals of these clinics. Based on the results of this study, it is suggested to managers to pay attention to the dimensions of the quality model of Donabedian (SPO) in setting up and developing the performance of HENLCs, it is recommended that future quantitative studies based on the categories formed in this study evaluate the observance of the dimensions of structure, process and outcome.

## Data Availability

All data generated or analyzed during this study are included in this published article.
